# Dual-Wavelength LiDAR with a Single-Pixel Detector Based on the Time-Stretched Method

**DOI:** 10.3390/s24175741

**Published:** 2024-09-04

**Authors:** Simin Chen, Shaojing Song, Yicheng Wang, Hao Pan, Fashuai Li, Yuwei Chen

**Affiliations:** 1 School of Computer and Information Engineering, Shanghai Polytechnic University, Shanhai 201209, China; 18656151701@163.com; 2 State Key Laboratory of Pulsed Power Laser Technology, National University of Defense Technology, Hefei 230037, China; ph_research2024@163.com; 3 Advanced Laser Technology Laboratory of Anhui Province, Hefei 230037, China; lifashuai@gmail.com (F.L.); chinaway.fgi@gmail.com (Y.C.)

**Keywords:** dual-wavelength LiDAR, NDVI, time-stretched

## Abstract

In the fields of agriculture and forestry, the Normalized Difference Vegetation Index (NDVI) is a critical indicator for assessing the physiological state of plants. Traditional imaging sensors can only collect two-dimensional vegetation distribution data, while dual-wavelength LiDAR technology offers the capability to capture vertical distribution information, which is essential for forest structure recovery and precision agriculture management. However, existing LiDAR systems face challenges in detecting echoes at two wavelengths, typically relying on multiple detectors or array sensors, leading to high costs, bulky systems, and slow detection rates. This study introduces a time-stretched method to separate two laser wavelengths in the time dimension, enabling a more cost-effective and efficient dual-spectral (600 nm and 800 nm) LiDAR system. Utilizing a supercontinuum laser and a single-pixel detector, the system incorporates specifically designed time-stretched transmission optics, enhancing the efficiency of NDVI data collection. We validated the ranging performance of the system, achieving an accuracy of approximately 3 mm by collecting data with a high sampling rate oscilloscope. Furthermore, by detecting branches, soil, and leaves in various health conditions, we evaluated the system’s performance. The dual-wavelength LiDAR can detect variations in NDVI due to differences in chlorophyll concentration and water content. Additionally, we used the radar equation to analyze the actual scene, clarifying the impact of the incidence angle on reflectance and NDVI. Scanning the Red Sumach, we obtained its NDVI distribution, demonstrating its physical characteristics. In conclusion, the proposed dual-wavelength LiDAR based on the time-stretched method has proven effective in agricultural and forestry applications, offering a new technological approach for future precision agriculture and forest management.

## 1. Introduction

As an active remote sensing technique, LiDAR (Light Detection and Ranging) is less affected by illustration conditions. It has the advantages of high spatial resolution, strong anti-interference ability, and high detection sensitivity [1]. Traditional single-wavelength LiDAR has been widely used in plot-level horizontal biomass measurement, canopy height profiles, and vertical forest structure in sample plots [2]. In addition to spatial 3D information, LiDAR can also obtain target intensity information at the selected wavelength of laser source, that is, the optical backscattered signal intensity, which is proportional to the total photon number incident on the detector [3]. Since the intensity is related to the reflectivity of the target at the given laser wavelength, it can reflect the characteristics of the target surface. However, the acquisition of target intensity information is affected by many factors, such as laser power, transmission distance, incidence angle, and target surface characteristics. Thus, the intensity information at a single wavelength cannot reflect the biochemical characteristics of the target effectively and straightforwardly [1]. Studies have shown combining spectral and LiDAR information can significantly improve the system’s capability for classification and extraction accuracy [4]. However, the direct fusion of active LiDAR measurement and passive multi-spectral/hyper-spectral imaging will result in temporal and spatial registration problems [5]. This problem prompted the development of multi-spectral/hyper-spectral scanners as single-data source solutions [6]. There is a strong demand that the spectral measurements from hyper-spectral imagers should be collected with spatial measurement from LiDAR simultaneously. Based on this, hyper-spectral LiDAR was developed to overcome the limitations of object recognition by providing registration-processing-free spectral and spatial information [7].

Hyper-spectral LiDAR can obtain 3D point clouds at different wavelengths and generate a colorful point cloud. This capability facilitates the achievement of high-precision and high-reliability classification for both targets and scenes [8,9]. So, it has attracted much attention from academic communities in recent decades. It is mainly used in forest and urban plant surveys [10,11,12], bathymetric mapping [3], topographic mapping [13], archaeology [14,15], geology [16], and navigation systems [17]. Researchers have recently considered multi-spectral LiDAR for developing an active spectral vegetation index (SVI) based on traditional optical classification [18]. The Normalized Difference Vegetation Index (NDVI) is one of the most widely used vegetation indices [19]. It is considered one of the best indicators of vegetation and background changes, especially in areas with dense vegetation [20]. Since the SVI can be associated with plant pigment concentration and leaf water content [21], the measurement of plant physiological characteristics and leaf water content can be realized by generating the vertical profile of the spectral index, as well as the separation of leaves and trees can also be realized [22,23]. Some progress has been made in implementing SVIs using multi-spectral LiDAR. Gong et al. designed a multi-wavelength canopy LiDAR (MWCL) system for vegetation reflection remote sensing. Four different wavelengths of lasers (556, 670, 700, and 780 nm) were used to retrieve vegetation characteristics, which improved the classification accuracy of vegetation canopies with similar structures [24]. Douglas et al. designed a ground-based full-waveform LiDAR scanner, the Dual-Wavelength Echidna^®^ LiDAR (DWEL), that can automatically retrieve forest structures, which uses 1064 nm and 1548 nm synchronous pulse lasers to separate leaf scattering from trunk, branch, and ground material scattering [25]. Zheng Niu et al. designed a 4-wavelength (531, 570, 670, and 780 nm) multi-spectral LiDAR prototype to monitor vegetation’s fine structure and biochemical parameters. Four avalanche diode modules (MenloSystems APD) were used as detectors. By constructing NDVI and photochemical reflectance index (PRI), it can detect the biochemical characteristics of vegetation [1]. Nikos et al. measured and analyzed apple position, quality-related size, and maturity-related chlorophyll using two LiDAR scanners (660 and 905 nm). From the 3D point clouds acquired by two LiDARs, the NDVI index was obtained. This method provides an effective tool for fine production management [26].

Multi-wavelength echo can obtain more intensity and spectral information so that the characteristics of the target can be refined. However, with the increase in wavelengths, the optical loss and demand for detectors will increase, making the system complex and expensive [27]. In order to meet the requirements of multiple wavelengths, the existing multi-spectral LiDAR uses multiple laser sources and photodetectors simultaneously. Vasanthi et al. developed a multi-spectral SWIR LiDAR system that simultaneously measures spatial-spectral information for imaging and spectral identification with partial occlusion. The system uses seven wavelengths for detection, corresponding to seven detectors [7]. With the development of laser sources, supercontinuum lasers provide the opportunity of tens or even hundreds of continuous spectral channels for multi/hyper-spectral LiDAR by generating white light sources. Compact systems are developed by combining light-splitting systems instead of multiple independent laser sources. Chen et al. designed a hyper-spectral LiDAR based on AOTF (Acousto-optic Tunable Filter) with a spectral resolution of 10 nm and a coverage range of 500–1000 nm to extract and evaluate vegetation parameters [28]. The system uses a single photodetector APD to achieve multi-wavelength detection. However, only a single wavelength of laser emission and detection is realized in each trigger of the laser source. Sun et al. proposed the use of an intelligent and miniaturized spectral element integrated optical filter (IOF) for the realization of a miniaturized hyper-spectral LiDAR. This system exhibits a spectral profile that is highly congruent with that of AOTF-based hyper-spectral LiDAR and demonstrates superior ranging capabilities. Furthermore, the miniaturized design of the IOF holds significant potential for specific applications, underscoring its promise for future technological advancements [29]. Kim et al. proposed a time division multiplexing-based multi-spectral LiDAR system that simultaneously acquires spatial and spectral information and uses a single optical detector to minimize optical losses. The system uses five lasers and a pulse clock to trigger different lasers to realize time division multiplexing [27].

In this research, we propose a time-stretched optical configuration to construct a multi-spectral LiDAR with low optical loss, complexity, and cost. This enables the efficient acquisition of vegetation indices to study vegetation’s physical properties. Based on the time-stretched method, the system uses a supercontinuum laser and a single detector to realize the detection of dual-wavelength or even more echoes. The design effectively saves the cost of the system and reduces the complexity. Combined with the laser equation for the scene analysis, we study the impact of the incidence angle on the target reflectance. The constructed system can collect two wavelength echoes in turn with single-pixel sensor under a single transmitting pulse simultaneously, and the incidence angle does not affect the NDVI, so the index can effectively characterize the physical characteristics of the target. Based on this principle, we test the ranging accuracy of the proposed system. NDVI acquisition tests were also performed for different targets to distinguish leaves, branches, and soil. Finally, combined with the motor-driven scanning system and acquisition equipment, a Red Sumach was scanned throughout to obtain its NDVI point cloud map, and different parts of the plant were visualized. The verification of the effectiveness and efficiency of the system shows that it will play an important role in the detection and precise management of agriculture and forestry.

This paper is organized as follows: Section 2 introduces the proposed time-stretched method and the constructed dual-wavelength LiDAR system. Section 3 explains the experiments, expounds on the need for calibration, and analyzes the results of ranging and NDVI experiments. Section 4 is devoted to the conclusion.

## 2. Materials and Methods

### 2.1. Time-Stretched Method

Based on the existing problems of multi-spectral LiDAR detection efficiency, we propose a time-stretched method to build a low-cost, low optical loss, and low complexity multi-spectral LiDAR system. This method divides the white light under a single pulse emitted by one supercontinuum laser into two different wavelengths—800 nm and 600 nm. For the 800 nm wavelength of the laser pulse, multiple reflections in space are used to generate a fixed time delay so that the laser of 800 nm wavelength leaves the transmitting system later than the laser of 600 nm wavelength and hits the identical target surface. Thus, the echoes reflected by the target arrive at the detector in turn. Therefore, efficient detection of multiple echoes can be achieved using only a single detector with a time-stretched setup.

### 2.2. System and Components

**Figure 1:**
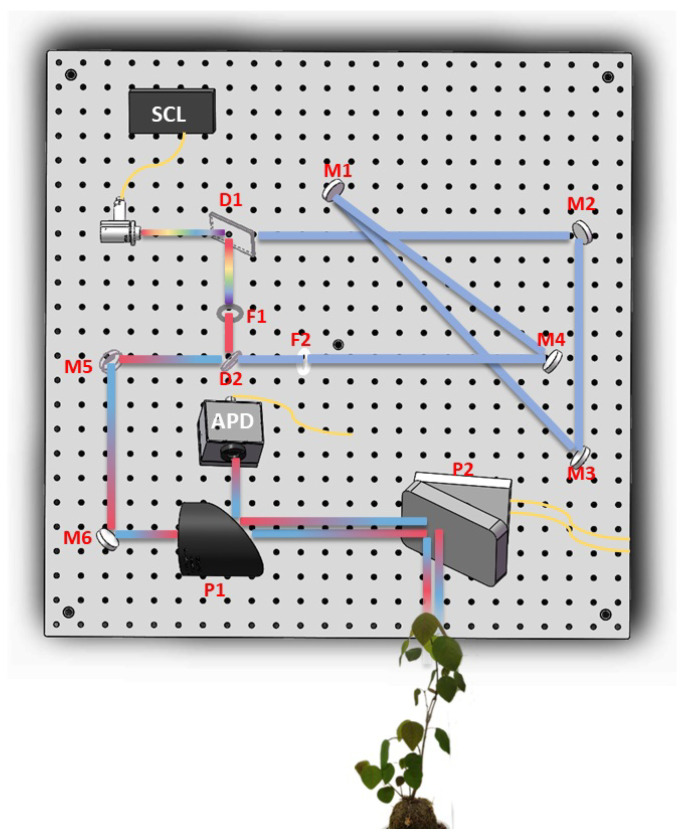
Diagram of the dual-wavelength multi-spectral LiDAR system architecture.

The dual-wavelength multi-spectral LiDAR that we constructed is shown in Figure 1. It consists of a laser source, a transmitting optical system, a receiving optical system and a collection device. A supercontinuum laser (LEUKOS SCM-30-HE-450) (Ricany, Czech Republic) was used as the light source to emit white light at a repetition frequency of 10 kHz. When the laser source enters the transmitting optical system, it is first divided into two paths by the dichroic mirror (Thorlabs, DMSP680B) (Newton, NJ, USA) (component D1 in Figure 1); one is the refracted beam of 705–1080 nm, and the other is the reflected beam of 400–660 nm. Beams in the 400–660 nm band directly reach the beamforming lens through a 600 nm bandpass filter, while beams in the 705–1080 nm band pass through four mirrors and then through an 800 nm bandpass filter to the beamforming lens. The light passing through the beamforming lens passes through 90° off-axis mirrors (Thorlabs, MPD339V-M01) (component P1 in Figure 1) and a planar mirror (component P2 in Figure 1) after leaving the transmitting optical system to reach the target. The two bands of laser light reflected off the target surface and then arrived at the receiving system and the photodetector APD (MenloSystems APD210) (Martinsried, Germany). We use an oscilloscope (Tektronix MSO64B) (Beaverton, OR, USA) with a 50 Gsps sampling rate for high-speed data acquisition. The characteristics of this dual-wavelength LiDAR is shown in Table 1.

#### 2.2.1. Supercontinuum Laser (SCL)

**Figure 2:**
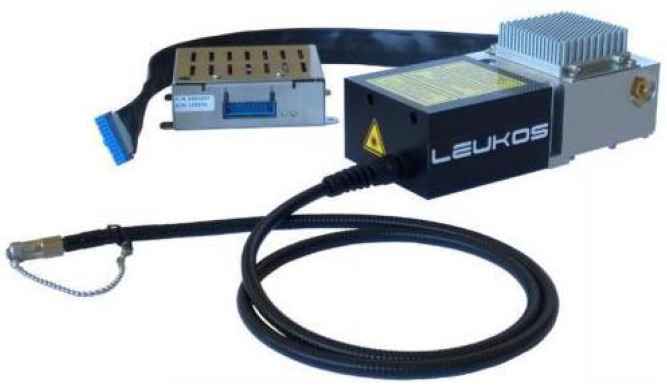
Physical image of the supercontinuum laser.

In order to make this multi-spectral LiDAR system scalable and highly tunable, we choose a supercontinuum laser to generate the laser. The LEUKOS laser (Figure 2) we use has an emission band of 200–2400 nm and a maximum power of 200 mW. The maximum energy of a single pulse is 19 μJ and a pulse width is less than 1.2 ns.

#### 2.2.2. Avalanche Photo Diode (APD)

The echo signal reflected by the target is very weak because of the absorption and scattering of the target. In order to detect the echo, it is also very important to select the appropriate detector. In order to obtain accurate range information for narrow pulse signals using the TOF (time of flight) ranging method, the detector response time needs to be fast enough. In LiDAR, PIN photodiode, APD, SPAD (single-photon avalanche diode) and PMT (photomultiplier tubes) are commonly used for photodetection of a weak echo signal.

**Figure 3:**
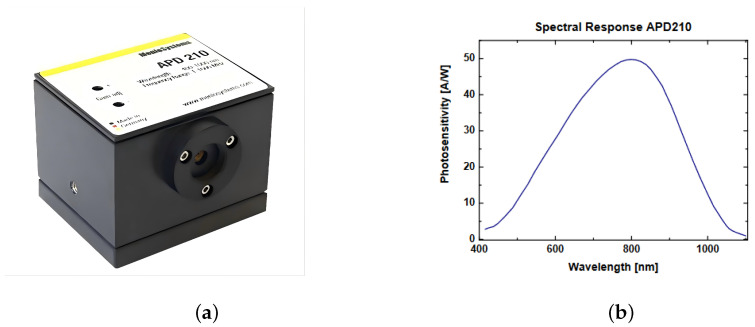
(**a**) Physical image of APD 210. (**b**) Spectral response curve of APD 210.

In this research, we select APD210 as the detector, which is a silicon avalanche photodetector. It can detect an echo spectral range from 400 to 1100 nm, with bandwidth from 5 MHz to 1 GHz and peak gain of 2.5 × $10^{5}$ V/W@800 nm, and its spectral response curve is shown in Figure 3b.

## 3. Experiment and Results

**Figure 4:**
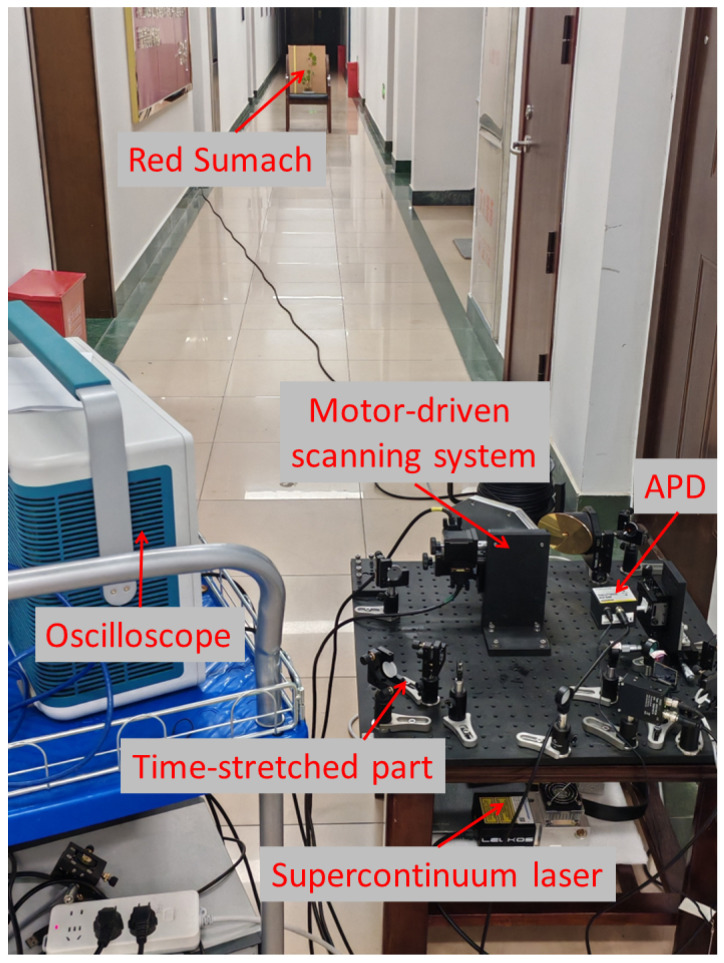
Dual-wavelength LiDAR demonstration instrument in the laboratory test.

Based on the dual-wavelength LiDAR system described in Section 2, we conducted experiments in the corridor of the experimental building as shown in Figure 4. The system initially collects the intensity values of the target, which then require calibration to be converted into target reflectance. Here, standard reflectance board (96% at 600 nm, 97% at 800 nm) were used to calibrate the intensity of leaves from Pachira Aquatica at different positions, demonstrating the necessity of calibration. In the ranging experiment, we still choose the leaf of Pachira Aquatica. By adjusting the leaf’s position, we measured the distance of each group according to time of flight (TOF), and then compared the variation with the data measured by the handheld range finder, so as to obtain the ranging accuracy of the system.

In practical scenarios, the orientation of the leaf may not be perfectly perpendicular to the laser beam, prompting an analysis of the impact of incident angles on target reflectance. Interestingly, changes in the incident angle did not affect the NDVI value. To showcase the system’s ability to capture a plant’s NDVI effectively, we selected different parts from Pachira Aquatica, including healthy green leaves, dry leaves, diseased leaves, green and yellow branches, and soil, as targets. Multiple detections were performed on these six types of targets to obtain their respective NDVI values. The results revealed that the NDVI of green leaves could be clearly distinguished from that of branches and soil. Building on theoretical principles and experimental evidence, we choose Red Sumach instead of Pachira Aquatica for scanning, thus illustrating the universality of the application. Its NDVI point cloud map was generated after data processing. This allowed for the visualization and discrimination of different parts of plants using this dual-wavelength multi-spectral LiDAR.

Totally, we conducted four comprehensive experiments, the details of which are outlined in Table 2. A thorough presentation of the experimental development, results, and subsequent analysis can be found in Section 3.1, Section 3.2 and Section 3.3.

### 3.1. Calibration

The radar equation has been applied to the LiDAR system [30]. This equation defines the received power as a function related to the system and sensor parameters. Since part of the laser light hitting the target surface is absorbed by the target, and the other part is scattered in all directions, the received optical power by the detector $P_{r}$ is as follows.

(1) $$P_{r}=\frac{P_{t}D_{r}^{2}}{4\pi R^{4}\beta_{t}^{2}}\sigma$$

where $P_{t}$ is the transmitted power, $D_{r}$ is the receiver optics’ aperture diameter, *R* is the range, and $\beta_{t}$ is the transmitter beamwidth. All target parameters are combined into one parameter, the backscattering cross section σ, which can be written as shown.

(2) $$\sigma =\frac{4\pi }{\Omega }\rho A_{s}$$

where Ω is the angle of the backscattering cone defined due to surface roughness, ρ is the reflectivity of the object, and $A_{s}$ is the receiving area of scattering.

Assuming that the spot falls completely on the target surface and the backscattering cross section is circular, $A_{s}$ can be defined as:

(3) $$A_{s}=\frac{\pi R^{2}\beta_{t}^{2}}{4}$$

At the same time, we assume that the target has a solid angle Ω of π, and the target is a Lambertian scatterer. Under these conditions, Equation (2) can be transformed into Equation (4).

(4) $$\sigma =\pi \rho R^{2}\beta_{t}^{2}cos\alpha$$

Here, the incident angle α is greater than zero.

According to Equations (1) and (4), the received signal power can be expressed as follows:

(5) $$P_{r}=\frac{P_{t}D_{r}^{2}}{4R^{2}}\rho cos\alpha$$

It can be seen from the Equation (5) that the size of the receiving surface, the reflectance and the incident angle jointly determine the backscattering characteristics of the target. In radar remote sensing, the measured receiver power is converted into the radar cross-section using the radar equation called calibration [30]. However, to use the intensity values collected by the LiDAR system to classify the target, only the calibrated data are meaningful [31]. The calibration is usually achieved by using the information of a reference target acquired by the system, which needs to have Lambertian scatterer characteristics and whose reflectance is known. Therefore, to obtain the real reflectance of the target collected by the LiDAR system, it is necessary to use a standard reflectance board for calibration [24]. We choose a standard whiteboard with known reflectance for calibration, and the received signal power of the reference target is as follows:

(6) $$P_{r,ref}=\frac{P_{t}D_{r}^{2}}{4R^{2}}\rho_{ref}cos\alpha$$

The reference target surface needs to be completely perpendicular to the laser beam to ensure the validity of the calibration. So, the angle of incidence is 0 degrees for the received signal power reflected from the reference target. However, in the scanning process, the actual target surface is not necessarily perpendicular to the laser beam, such as the leaf normal vector of the plant pointing in any direction in the space. Therefore, for the received signal power reflected from the target, the incidence angle is between 0 and 90 degrees, leading to low received signal power. In the actual scenario, the received signal power of the target and the reference target at the distance $R_{i}$ are as follows:

(7) $$\left\{\begin{array}{c} P_{r_{i}}=\frac{P_{t_{i}}D_{r}^{2}}{4R_{i}^{2}}\rho_{target}cos\alpha \\ P_{r_{i},ref}=\frac{P_{t_{i},ref}D_{r}^{2}}{4R_{i}^{2}}\rho_{ref} \end{array}\right.$$

The transmitted laser power is assumed to be the same for the target and the reference target. According to Equation (7), the target reflectance can be obtained as follows:

(8) $$\rho_{target}=\frac{1}{cos\alpha }\cdot \frac{P_{r_{i}}}{P_{r_{i},ref}}\rho_{ref}$$

From Equation (8), it can be seen that the reflectivity of the actual target acquired during scanning will be larger than that of the target whose surface is completely perpendicular to the laser beam.

**Figure 5:**
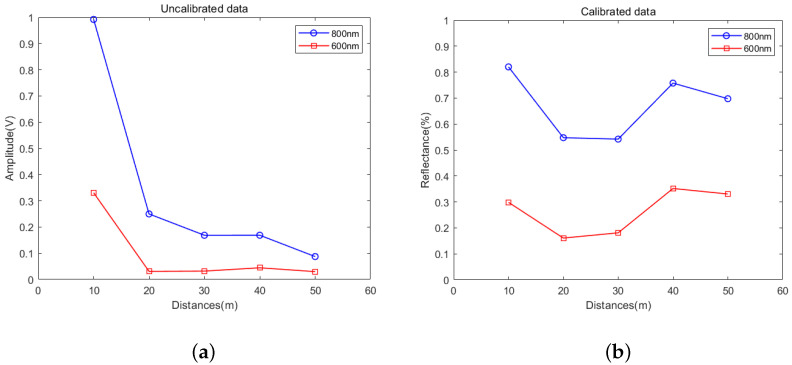
(**a**) The intensity of leaf collected by the system at distances of 10 m, 20 m, 30 m, 40 m, and 50 m; (**b**) is the corresponding reflectance calibrated by a standard SRB.

We placed the leaf from Pachira Aquatica and the standard reflectance board (SRB) at distances of 10 m, 20 m, 30 m, 40 m, and 50 m from the laser source, respectively. It is also ensured that both the target and reference target surfaces are perpendicular to the laser beam, respectively, so that the incidence angle does not affect the calibration results. The intensity values of the whiteboard and leaf at each position were collected 5 times, and the final intensity value of the position was obtained by averaging. The change curves of leaf echo intensity at 800 nm and 600 nm wavelengths are shown in Figure 5a. Accordingly, the reflectivity change curve after calibration with the SRB is shown in Figure 5b.

**Figure 6:**
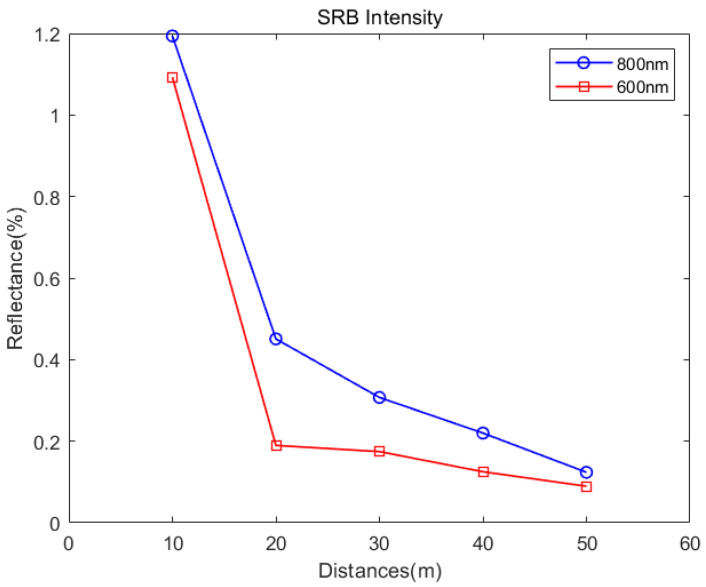
The intensity of SRB collected by the system at distances of 10 m, 20 m, 30 m, 40 m, and 50 m.

It can be seen that the echo intensity of the two wavelengths at 600 nm and 800 nm decreases with the increase in range. The intensity value decreases sharply when the distance increases from 10 m to 20 m. It reduces from 0.99 to 0.25 under the wavelength of 800 nm. Simultaneously, it reduces from 0.33 to 0.03 under the wavelength of 600 nm. However, beyond 20 m, the intensity does not change much. From 20 to 50 m, the leaf echo intensity at 800 nm wavelength decreases by 0.16 to 0.09, and the intensity at 600 nm wavelength only decreases by 0.01. The attenuation law of light causes the change. From Figure 6, it can be seen that the variation in the intensity of SRB with distance is consistent with that of the leaf.

After calibration, the variation range of reflectance is reduced. Following conversion of the echo intensity at 800 nm wavelength to reflectance, the fluctuation range is 0.28. Similarly, the echo reflectance variation range at 600 nm wavelength is only 0.19. This is much smaller than the original intensity value because the reflectance obtained after calibration demonstrates the physical characteristics of the target and will be barely affected by distance. The reason for the fluctuation is that when moving the leaf, the spot cannot be guaranteed to fall in the same position as before, and the difference in physical characteristics between different positions of the leaf will lead to certain variations in the results.

### 3.2. Ranging

To evaluate the ranging performance of the system, we tested its ranging accuracy. The experiment is designed as follows:

1. The leaf from Pachira Aquatica is placed at a distance of more than 10 m from the laser source, then 5 echoes are acquired to calculate the range and average them as the first distance;
2. The position of the leaf is adjusted to about 15 cm back, referring to the initial position, then the second distance is obtained in the same way with 1;
3. Refer to 2 to obtain the distance information of leaves under five positions.

The distance is calculated with TOF (time of flight). We take the trigger time as the start time $t_{start}$ and the echo peak as the stop time $t_{stop}$. Both time signals are acquired by the oscilloscope. Among them, the start time is when the SCL emits the laser pulse, the signal is simultaneously transmitted to the oscilloscope through the synchronous trigger interface, and the oscilloscope is triggered to start the acquisition. The stop time corresponds to the time when the echo peak is located. Combined with the speed of light *c*, the target distance *d* is calculated as follows:

(9) $$d=\frac{c}{2}t_{stop}-t_{start}$$

Finally, the distance information at five positions measured by the system is shown in Table 3. We measured each position with a handheld range finder to obtain the reference range information.

Comparing the distance measured by the system with the reference distance measured using the range finder, the average error is 0.18 m. To further illustrate the effectiveness of the ranging of this system, we calculate its relative error as shown in Table 4. The distance variation is calculated as the difference between the distance of the target at two adjacent transformed positions, as Equation (10). This is because the comparison between the range information obtained directly by the LiDAR system and the range finder is greatly affected by human factors, and the error value obtained deviates from the actual system performance. Therefore, we convert the distance data directly obtained by the LiDAR system and the range finder to obtain the distance variation, and then evaluate the ranging accuracy of the system according to the relative error of the variation. This method of assessment is relatively objective.

(10) $$d_{variation1}=d_{position2}-d_{position1}$$

It can be seen that the average relative error is about 3.2 mm, and the reasons are as follows:

1. The sampling rate of the oscilloscope is 50 Gsps, so the interval of sampling points is 20 ps. The theoretical ranging resolution calculated by the speed of light is 3 mm. The corresponding relative error of measurement is about 3 mm;
2. Using the TOF method to calculate distance, the determination of start time and stop time is very important for the accuracy of distance measurement. The error of time will lead to the generation of distance error. When the system is used to measure distance, the start time is determined by the trigger time, and the trigger signal will have a certain offset and jitter, affecting the accuracy of time determination;
3. Our target object is a foam plate with a leaf glued to it, and the plane placed on it is not completely perpendicular to the laser beam, which will also lead to errors.

### 3.3. NDVI

#### 3.3.1. Description of NDVI

Kriegler et al. [32] proposed a simple band transformation in 1969: near-infrared (NIR) radiation minus red radiation divided by near-infrared radiation plus red radiation, and the normalized vegetation index (NDVI) can be obtained. This index was initially acquired through remote sensing techniques for analyzing vegetation information, achieving the differentiation of land types, and estimating various vegetation properties such as LAI (Leaf Area Index), biomass, leaf chlorophyll concentration, vegetation coverage, etc.

We use this dual-wavelength LiDAR to generate laser light at 800 nm and 600 nm wavelengths to calculate NDVI using the following formula [33]:

(11) $$NDVI=\frac{\rho_{800}-\rho_{600}}{\rho_{800}+\rho_{600}}$$

Here, $\rho_{800}$ and $\rho_{600}$ represent the target reflectance at 800 nm and 600 nm wavelengths, respectively.

We know from Section 3.1 that the incidence angle affects the target reflectance magnitude. In the scanning process, we cannot obtain the leaf angle where each spot is located, so it is necessary to select an appropriate index to reflect the real physical characteristics of the target. According to Equation (8), we obtain the target reflectance at 800 nm and 600 nm wavelengths as follows:

(12) $$\left\{\begin{array}{c} \rho_{800}=\frac{1}{cos\alpha }\cdot \frac{P_{r_{i},800}}{P_{r_{i},ref,800}}\rho_{ref,800}\\ \rho_{600}=\frac{1}{cos\alpha }\cdot \frac{P_{r_{i},600}}{P_{r_{i},ref,600}}\rho_{ref,600} \end{array}\right.$$

Combining Equations (11) and (12), the NDVI can be obtained as follows:

(13) $$NDVI=\frac{\frac{P_{r_{i},800}}{P_{r_{i},ref,800}}\rho_{ref,800}-\frac{P_{r_{i},600}}{P_{r_{i},ref,600}}\rho_{ref,600}}{\frac{P_{r_{i},800}}{P_{r_{i},ref,800}}\rho_{ref,800}+\frac{P_{r_{i},600}}{P_{r_{i},ref,600}}\rho_{ref,600}}$$

Since the system uses a single APD to detect the dual-wavelength echoes simultaneously based on the time-stretched method, the incidence angles corresponding to the two wavelengths are the same. The final NDVI is not affected by the incidence angle and can directly characterize the physical characteristics of the target.

#### 3.3.2. Comparison of Different Objects’ NDVI

**Figure 7:**
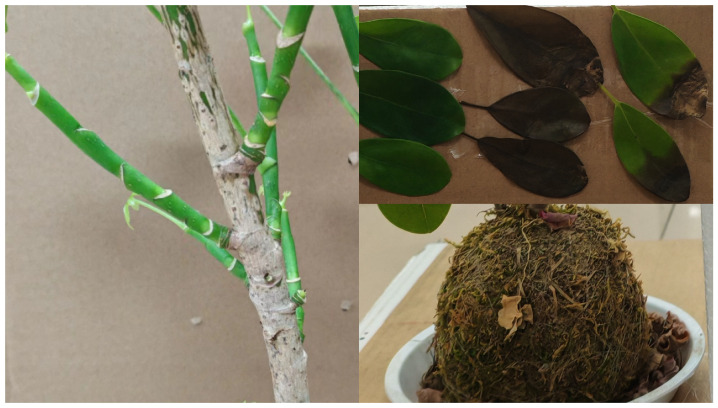
Photos of green leaves, dry leaves, diseased leaves, branches, and soil were selected in the experiment.

Since leaves’ water content and chlorophyll concentration in different statuses, branches, and soils are different, the NDVI reflecting their physical characteristics will also be varied. In order to observe whether there are obvious NDVI differences among them and verify the system’s effectiveness, we selected the following targets for detection: green leaves, dry leaves, diseased leaves, branches from Pachira Aquatica and soil (Figure 7). To ensure the health status of the green leaves, we measured them directly after picking them from the plant body. Because the diseased leaf is partly pathological, we measured the healthy and diseased parts. The branches were observed to have green and yellow branches in terms of color, and we measured both.

**Figure 8:**
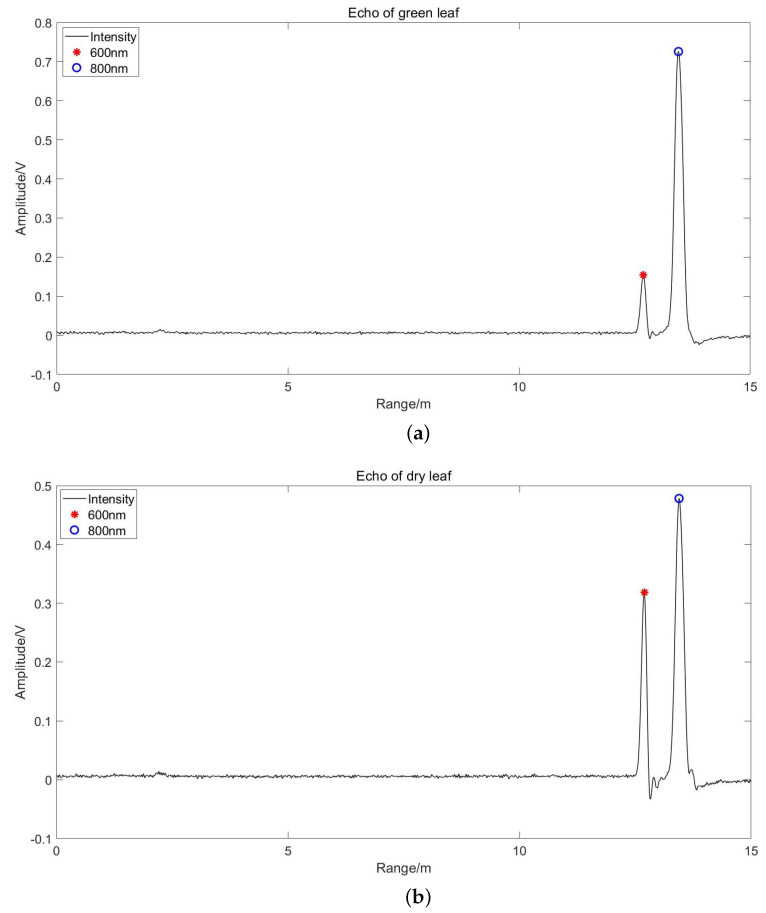

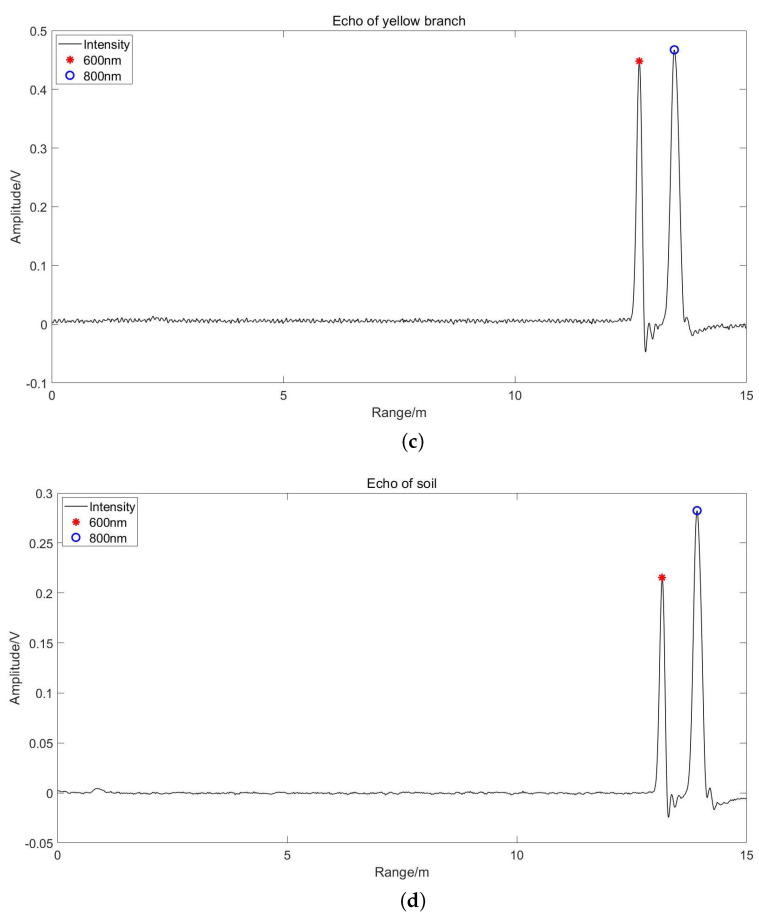
Echo waveform of (**a**) green leaves, (**b**) dry leaves, (**c**) yellow branches, and (**d**) soil.

We put the target at a fixed position and collected 10 groups of echoes for each target. Using the data collected by the oscilloscope, we processed and obtained the intensity corresponding to 600 nm and 800 nm, as shown in Figure 8. Then, intensity can be converted to reflectance according to the calibration method of Section 3.1 to calculate the NDVI of each data group. The final NDVI value of the target is the average result of ten data groups.

**Figure 9:**
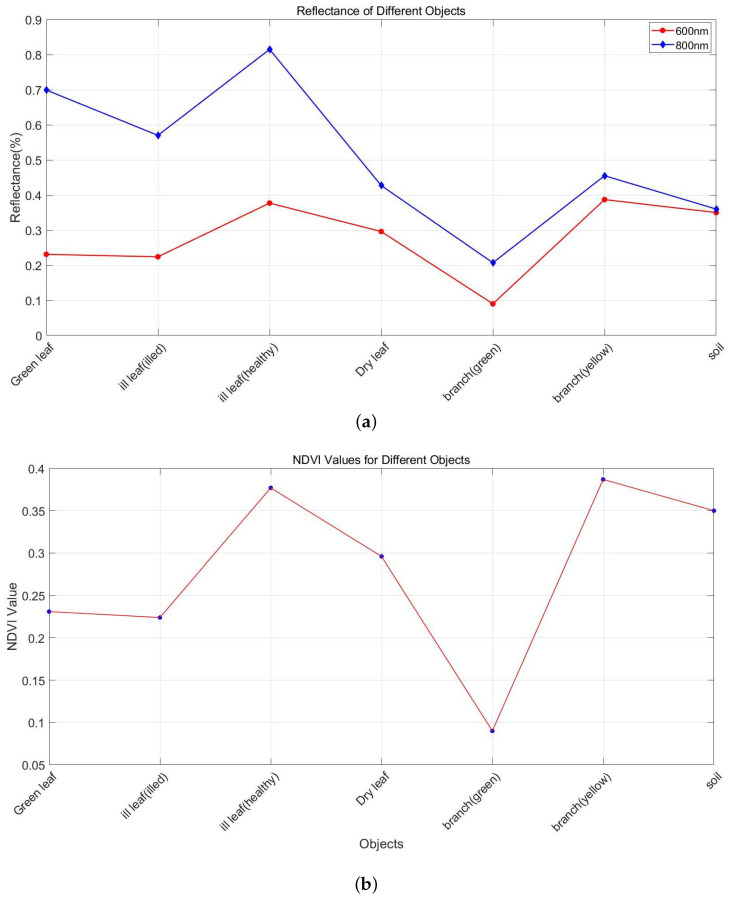
(**a**) Reflectance at 800 nm (blue) and 600 nm (red) as well as (**b**) NDVI of green leaf, ill leaf (the unhealthy part), ill leaf (the healthy part), dry leaf, green branch, yellow branch, and soil.

We present the NDVI values of different targets in Figure 9. It can be seen that the NDVI value of healthy green leaves is the highest, 0.518, which is due to the high water content and high chlorophyll content of healthy green leaves. On the contrary, the NDVI of dry leaves was only 0.18. The NDVI value of diseased leaves was between green leaves and dry leaves, and diseased parts were higher than that of healthy parts. This is because the water and chlorophyll content of diseased leaves were partly lost but still higher than that of dry leaves. The low discrimination of diseased leaves may be because different leaves have different diseases, and the main elements are not sensitive to NDVI. The NDVI of green branches was almost equal to that of leaves (0.391), while the NDVI of yellow branches was almost similar to that of soil (0.08). The NDVI of the soil is almost close to 0, which is consistent with the reality.

The NDVI plots in Figure 8 show that the NDVI values of green and dry leaves are quite different. The NDVI values of soil and branches are relatively close, and both tend to be 0. This is important for distinguishing leaves in healthy plants, branches, and ground.

#### 3.3.3. Cloud of Points

Based on the radar equation, the deviation of the incidence angle does not affect the magnitude of the NDVI of the target in the actual scene. Simultaneously, by collecting the reflected echoes of different targets and analyzing their reflectance and NDVI, it is found that NDVI can effectively reflect the target’s physical characteristics, mainly water and chlorophyll. Based on these, we used this system to scan a Red Sumach and obtain its point cloud map. The purpose of choosing Red Sumach instead of Pachira Aquatica here is to demonstrate the universality of this system. This is firstly because the morphology of Red Sumach is similar to that of most agroforestry plants. Moreover, most of the leaf surface is in the vertical plane of the laser incident direction, which makes the visual effect of the obtained point cloud image better and makes the result analysis more intuitive. Secondly, we use the Pachira Aquatica here to obtain NDVI comparison of leaves, branches, and soil in different statuses, and then use Red Sumach to generate the scanned NDVI point cloud map of it to realize the visualization of different parts, which can effectively confirm the results of NDVI comparison in the previous stage, and also illustrate the universality of this system.

**Figure 10:**
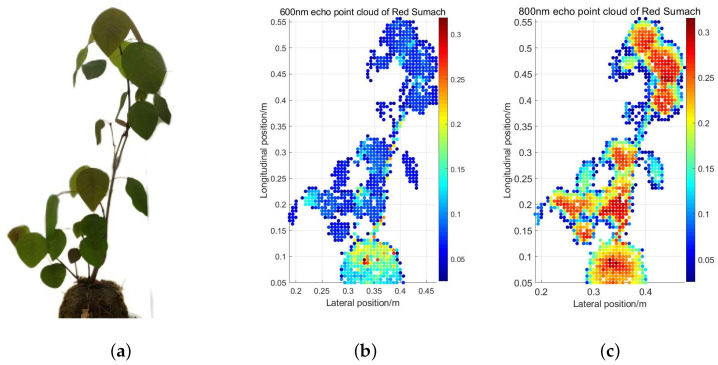
(**a**) Photo of Red Sumach; (**b**) 600 nm echo point cloud of Red Sumach at 10 m; (**c**) 800 nm echo point cloud of Red Sumach at 10 m.

Since 800 nm is delayed by 2.5 ns relative to the 600 nm wavelength, the distance between the target plant and the background should be less than 76 cm to avoid the interference of the secondary echo to different wavelengths. The distance between our background and the target in the experiment is about 35 cm. The single laser pulse triggered by the SCL is launched through the transmitting optical system shown in Figure 1, and the laser beams with 600 nm and 800 nm wavelengths are separated in the time dimension using the time-stretched method. The laser beam is reflected back to the receiving system through the target surface, and the echoes of the two wavelengths are detected and received by the APD in turn, then transmitted to the oscilloscope for acquisition. Under the control of the motor, after completing the data acquisition at each position, the angle of the plane mirror will be adjusted and the system will conduct the echo acquisition at the next position. According to the set starting position, ending position, and stride, the motor cooperated with the optical path system to complete the scanning of the plant. We use the collected data and the motor angle data to obtain the three-dimensional position information of the target after processing. For the removal of background, the threshold method is used to process the longitudinal distance information. We use the intensity value of each location to represent the point’s color, which is convenient for observation and analysis. Specifically speaking, we rotate the generated 3D target point cloud map to obtain the Red Sumach’s point cloud map in the X–Y plane. The reflectance of each point of the target at 600 nm and 800 nm wavelengths is presented in different colors in the Figure 10b and Figure 10c, respectively. The colorbars illustrate the reflectance of the target points corresponding to different colors. From the point cloud of 600 nm red light, plants and soil can be distinguished, but the difference is not visible at 800 nm. In order to see the distinction of physical characteristics between different parts of Red Sumach more intuitively, we draw the NDVI point cloud map. The intensity data at the same horizontal position are processed, and the NDVI is obtained according to the reflectance of the two wavelengths. The point cloud is as Figure 10.

**Figure 11:**
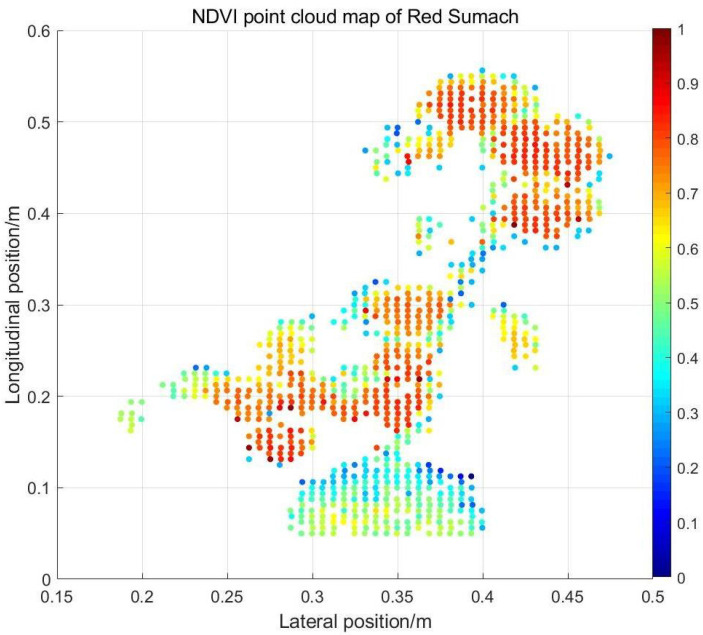
NDVI point cloud map of Red Sumach.

Combining the reflectance of each point of the target at 600 nm and 800 nm, we plotted the NDVI point cloud of Red Sumach. Accordingly, the NDVI value of each point is represented by different colors. As is shown in Figure 11, the NDVI point cloud map shows that the NDVI index of the leaf part is higher, and is closer to 1. However, the NDVI index of soil parts and branches is even lower and biased towards 0. This is consistent with the previous results of single-point acquisition data for different targets. The NDVI value of the leaf edge part will be lower than that of the middle part of the leaf due to the low water content. At the same time, in the edge zone, it is easy to generate the second echo due to the occlusion. Partial footprint falls on the front leaf, and the rest falls on the background or the back leaf. If partial footprint falls on the background, it will not affect the obtained NDVI of green plants because we only select the first echo data at this wavelength. However, if a partial footprint falls on the back leaf, the two echoes will overlap due to the close distance, resulting in a smaller NDVI acquisition. This might be the reason that why NDVI in the leaf edge zone is smaller than in the leaf center.

## 4. Conclusions

This paper proposes a dual-wavelength multi-spectral LiDAR system based on the time-stretched setup. The 600 nm wavelength laser and the 800 nm wavelength laser are interleaved in space by using this method so that one of the wavelengths is delayed a specific period in the time domain, and then echo acquisition of two wavelengths can be realized by a single APD. Based on this method, we realize the detection and reception of two wavelength echoes of a single pulse emitted by a laser with a single detector. Compared with the existing system, the constructed multi-spectral LiDAR system saves system’s costs and hardware space and improves acquisition efficiency. Specifically, the first is the cost of the system. Compared with the two-wavelength LiDAR in [25,34], we use the time-stretched method to reduce the number of lasers and detectors to one. Despite the increased number of mirrors, this is low compared to the reduced cost of transceiver devices. The second is the detection efficiency, because the detection and acquisition of dual-wavelength echoes can be realized under a single laser pulse. Therefore, compared with the LIDAR system in [15,16], which collects echoes of different wavelengths one by one pulse, the acquisition efficiency is significantly improved. At the same time, we use SCL to generate lasers with a wide band range, so compared with the systems in [9,24] that use fixed wavelength lasers, our system is more flexible and scalable. We verify the constructed system’s ranging performance and echo effectiveness, knowing its ranging accuracy can reach 3 mm. The intensity information of the echo is calibrated to obtain the reflectance. The echo information of leaves in different states, branches, and soil is processed separately to obtain the respective NDVI, which can observe the differences between the physical characteristics of leaves, branches, and soil. Based on theoretical justification and experimental analysis, we scanned Red Sumach at 10 m to obtain its NDVI point cloud maps, separating leaf parts from branches and soil parts could be achieved. It shows that the system can be further applied in agriculture and forestry.

In the future, we will expand the number of wavelengths to obtain more physical characteristics of the target. Additionally, we plan to collect and process echo data from various plants under different environmental conditions to broaden the application scope of our system. Furthermore, its application field will be extended, and the corresponding wavelength will be selected based on different application scenarios, such as mineral exploration and archaeology, to efficiently acquire target information. Our acquisition equipment will also be further optimized to realize the miniaturization of the system.

## Figures and Tables

**Table 1:** Specifications of the dual-wavelength LiDAR based on the time-streched method.

Parameters	
Laser wavelength	600 nm; 800 nm
Pulse repetition Frequency	10 kHz
Spectral width	40 nm
Beam divergence	0.07 mill rad
Beam diameter (at exit)	10 mm

**Table 2:** A brief description of each experiment.

Experiment	Materials	Methods and Objective
Calibration	Green leaf of Pachira Aquatica; SRB	Collect leaf’s intensities at five positions and use SRB to calibrate. This experiment is to convert intensity into reflectance and illustrate the necessity of calibration.
Ranging	Green leaf of Pachira Aquatica	Adjust the leaf’s position and measure the distance of each group according to TOF, then compare the variation with the data measured by the handheld range finder. This experiment is to obtain the ranging accuracy.
Different objects’ NDVI	Healthy green leaves, dry leaves, diseased leaves, green and yellow branches of Pachira Aquatica, soil; SRB	Acquire echoes from each target multiple times, calibrate the data, calculate the respective NDVI, and compare the results. This experiment is to show the effectiveness of this LiDAR system to obtain plant’s NDVI.
Cloud of points	Red Sumach; SRB	Control the LiDAR system to scan Red Sumach at 10 m, and process the data to obtain their NDVI point cloud map. This experiment is to prove that this LiDAR system can effectively visualize different parts of the target plant.

**Table 3:** Ranging result of the leaf at 5 distances.

System Measured Distance/m	Range Finder Measured Distance/m	Error/m
12.8538	12.6760	0.1778
13.0080	12.8310	0.1770
13.1520	12.9798	0.1722
13.3224	13.1442	0.1782
13.5204	13.3430	0.1774

**Table 4:** Relative error of ranging.

System Measured Distance Variation/m	Range Finder Measured Distance Variation/m	Relative Error/m
0.1542	0.1550	0.0008
0.1440	0.1488	0.0048
0.1707	0.1644	0.0063
0.1980	0.1988	0.0008

## Data Availability

The data that support the findings of this study are available from the corresponding author, sjsong@sspu.edu.cn, upon reasonable request.

## Abbreviations

The following abbreviations are used in this manuscript:

LiDAR	Light detection and ranging
NDVI	Normalized difference vegetation index
SVI	Spectral vegetation index
MWCL	Multi-wavelength canopy LiDAR
DWEL	Dual-wavelength echidna^®^ LiDAR
PRI	Photochemical reflectance index
AOTF	Acousto-optic tunable filter
IOF	Integrated optical filter
SCL	Supercontinuum laser
APD	Avalanche photo diode
SPAD	Single-photon avalanche diode
PMT	Photomultiplier tubes
TOF	Time of flight
SRB	Standard reflectance board
LAI	Leaf area index
